# Supplementary material for: Patterns of Intron Gain and Loss in Fungi
Source: PLoS Biol. 2004 Nov 30;2(12):e422. doi: 10.1371/journal.pbio.0020422 (PMC532390; doi:10.1371/journal.pbio.0020422)
Supplement: Table S1 — Also available at http://genes.mit.edu/NielsenEtAl/. (4.3 MB ZIP). [file pbio.0020422.st001.zip › NielsenEtAl/html/571.html]

AN4170.1.NCU03887.1.MG01045.1.FG08448.1


```
 CLUSTAL W (1.82) Multiple Sequence Alignments - Introns Inserted


Sequence 1: NCU03887.1	626 aa
Sequence 2: FG08448.1	596 aa
Sequence 3: MG01045.1	644 aa
Sequence 4: AN4170.1	597 aa
Alignment Length: 672 aa
Number Identitical Residues: 165 aa
Alignment Score (without introns) 9501


MG01045.1 	MPSFNPFSAVTGRHAFSLFDIR2LENDFLVFRGNEHEAASQLLKGVLAVCVPGPTRIEDI
NCU03887.1	MSFTNFFTSVTGKHAYTYFDIR2LESDFIVFRGNEHESAGQLLQGTVVLCLPAPLKIEDV
FG08448.1 	MPSFKPFASVTGRNSYSLFDIR2LDSDFIVFRGSDHESSGQILKGTLVLCLQSSLKIEDV
AN4170.1  	-MALSFFSGGGSASHAKYFDIR2LDEDYIVFRGGEQEAASAHLSGKLVLCVSEPISIKHI
          	    . *:.  .    . **** *:.*::****.::*::.  *.* :.:*:  .  *:.:

MG01045.1 	HLRLIGTLRLS2WADTKATPSGISIQKFEKTTEILSHRWPSFIGTPG-------------
NCU03887.1	HLRLTGTLHMS2WNDPRVTAAGISNHKIDRTSTIYSHRWKPFVGVGAENQSIGSPNGLMS
FG08448.1 	RLRLVGTLRHV2WADPRGTAPGVSGQKVDKTTTILEHRWAPFIGTHG-------------
AN4170.1  	RLHLTGISRVC2WHLPSSSAGGG--RKNWRERVFYEKTWK-FRDAGK------------S
          	:*:* *  :   *  .  :. *   :*  :   : .: *  * ..              :

MG01045.1 	KSMILDKGNYEFPFELMLPGNTSESVEGLKEASVTYRLKATIARGKLAYDHHAYKHIRMI
NCU03887.1	RGVTLPAGNYEWPFELMLPGDMTESVEGLREASLTYKLKATVARGKLAYDLHAYKRLRII
FG08448.1 	KSMTLPAGNYEWPFEYMLPGNMAESVEGIPEASITYRLRATVGRGKLAYDLHANKHLRII
AN4170.1  	KTEILPAGNYEYPFDVILEGSMPESVEGLSDTYVTYRFKAEIGR-KYAKDIVVRRPLRII
          	:   *  ****:**: :* *. .*****: :: :**:::* :.* * * *  . : :*:*

MG01045.1 	RTLDASALEFLHAM~SVENIWPNKVEYSIVVPRKAVVFGSAIPFESRFTPLLKGLEIGDV
NCU03887.1	RTLDPSTLEFLHTM~SVENIWPNKIEYSIMIPKKAVVFGSTIPLQTRFTPLLKGLELGEI
FG08448.1 	RTLEPGALEFLHAM~SVENIWPNKVDYSIIIPQKAVVFGGLVTMEMRFTPLLKGLELGDI
AN4170.1  	RTLESSALELSHAM0SVENIWPNKIEYSISTPTKAVIFGTSIRVDFKLIPLLKGLGIGQI
          	***:..:**: *:* *********::***  * ***:**  : .: :: ****** :*::

MG01045.1 	TIRLIEACEILATS-ATGVPIKEHKKEREVTSWVLPVTRDEHWQDVIENTGQEGWVLTAD
NCU03887.1	TIRLLEVHEFILQS-HSGYGVREHKKEREISVWKIPIEREKHWQDVIEDTGQEGWVMNTG
FG08448.1 	TARLMEVRECYVQASSSGHGTREHRTEREVALWKFAASREEHWQDMIEDTGQEGWALTKK
AN4170.1  	ISQLIETHDLTLNPEDPDAIRNTYKTTRTIINDEHTID-EENSLEIIDEAA-EGFQFSRT
          	  :*:*. :    .. ..   . ::. * :     .   :::  ::*:::. **: :.  

MG01045.1 	LDLPRKLGKCVQDVNTHGIKTRHKLKIVVALQNPDGHISE0LRATLPVTIFISPNIPLDE
NCU03887.1	LDLPRTLGKCVQDVNAHGIKVRHKLKVVLALHNPDGHISE~LRATLPVSIFISPNMPLDE
FG08448.1 	LNLPKRLRQCIQDLNHHGIKVRHKIKLTVALCNPDGHISE0LRATLPVSIFISPNMPLDE
AN4170.1  	LDLPKTLTRCLQDTDTRGIKVRHKLKFRVQLLNPDGHISE0LRATLPVSIFISPNLAIDD
          	*:**: * :*:** : :***.***:*. : * ******** *******:******:.:*:

MG01045.1 	DGNLAQPV-QQNSSSQESTSQIAPPGYGEHVLDQLYSNIDMAGYQTPGVQSGMNSPYMAL
NCU03887.1	EGNLVRQM-PNG-TTPGDVVAAPPPSYDEHTLDQLYEDMEPTGLQTP---AGMASPLYGH
FG08448.1 	EGNLVDQS-PTTPNNERELSTIAPPGYGEHVLDQLYENVDVSGFQTPGFQSGVSSPFYAQ
AN4170.1  	NNNLVDSSPQTTQRALDDLAQQAPPLYGEHQFDQLYSEVDPSGYRTPGPGSGPGTPFGTL
          	:.**.   .        .    .** *.** :****.::: :* :**.  :*  :*    

MG01045.1 	SRAGSNENLAAMLNDN--TITPAALTSRLQDVSVAGGPPRRDSSYASL--ASALLPTQNA
NCU03887.1	SRAGSVENLAALLHMHSTAVPPAALTSRLQNVSLE--PSSRSTSWNDG--DSETATPNNG
FG08448.1 	SRAGSNENLAALAHSR--PVAPAALSSRLANVSLD--PSQRTSSYTSL--HSAGSPGFMS
AN4170.1  	SRNLSAENLASMNAITHTDISASALHHRLVNLDLRGHGRVSASEHDHLGVPSDNGPPSGS
          	**  * ****::     : :..:**  ** ::.: .      :.    .  *   .   .

MG01045.1 	DTTPANTSPPASAPLSRHDSG----EENHSGNQTPEHVDFPELSELSKVPSYATAIRSSR
NCU03887.1	PADSVHSSAFPSAPLTRQNSGDNITAEAMSGYHTPEHLDFSGIMELSKVPSYHTAVKTPV
FG08448.1 	PTSAPQYQAPRSEPVTAALTRTNSSEDSSRNSAEHVDLDATDLAELSKVPSYQTAVKTPA
AN4170.1  	NTHGSNTHAPGSPELSRRASDEDVHDNIPSGMATPFIPHSAELETLSRVPSYSTAVRSSV
          	 :   :  .  *  ::   :  .   :   .       . . :  **:**** **:::. 

MG01045.1 	--VFAPAANALPDYATATSEPTTPAVTTPVMTPVAQTANPLELIPEDPLEQTGAGSVSPA
NCU03887.1	RPIAYPEGTVLPDYVAATSASGS---------------------------------TTPG
FG08448.1 	RSRAGTGDLLLPDYQTALSAP-----------------------------------RTPP
AN4170.1  	R----PHDSDLPDYQAVVAETVH--------------------------------MSAPQ
          	     .    **** :. : .                                    :* 

MG01045.1 	ATPVSPSTMSTS---ADQHHNHHHHNPQRPTPRRHSSFGGFFSSHHSLGMHHFHHHHGEG
NCU03887.1	SPELGHSEAGDGASKVEERNQSRPEPPRRKSARSRLAFTLSHIPHIHHSHSHHGHSHSES
FG08448.1 	ATDLAPDPLGT----ISEDQDGEDHTQHISRPASWIAG--------GRAYTDDLASYRRS
AN4170.1  	SPQQAHIRGSG---------TGRGSDSYFSAPMDFFHR--------PAFLHSRSHSHSDD
          	:.  .    .            .        .                        :  .

MG01045.1 	DERRRFTLMQMRDPVS
NCU03887.1	EDRRRHSSMMQAN---
FG08448.1 	QNRNQVF---------
AN4170.1  	ERRIRLTQARGRA---
          	: * :
```
